# Surveillance study of vector species on board passenger ships, Risk factors related to infestations

**DOI:** 10.1186/1471-2458-8-100

**Published:** 2008-03-27

**Authors:** Varvara A Mouchtouri, Rimma Anagnostopoulou, Anna Samanidou-Voyadjoglou, Kalliopi Theodoridou, Chrissi Hatzoglou, Jenny Kremastinou, Christos Hadjichristodoulou

**Affiliations:** 1 Department of Hygiene and Epidemiology, Medical School, University of Thessaly, Larissa, Greece; 2 Department of Public Health, Prefecture of Piraeus, Piraeus, Greece; 3 Department of Parasitology, Entomology & Tropical Diseases, National School of Public Health, Athens, Greece; 4 Department of Public and Administrative Health, National School of Public Health, Athens, Greece; 5 Department of Physiology, Medical School, University of Thessaly, Larissa, Greece

## Abstract

**Background:**

Passenger ships provide conditions suitable for the survival and growth of pest populations. Arthropods and rodents can gain access directly from the ships' open spaces, can be carried in shiploads, or can be found on humans or animals as ectoparasites. Vectors on board ships may contaminate stored foods, transmit illness on board, or, introduce diseases in new areas. Pest species, ship areas facilitating infestations, and different risk factors related to infestations were identified in 21 ferries.

**Methods:**

486 traps for insects and rodents were placed in 21 ferries. Archives of Public Health Authorities were reviewed to identify complaints regarding the presence of pest species on board ferries from 1994 to 2004. A detail questionnaire was used to collect data on ship characteristics and pest control practices.

**Results:**

Eighteen ferries were infested with flies (85.7%), 11 with cockroaches (52.3%), three with bedbugs, and one with fleas. Other species had been found on board were ants, spiders, butterflies, beetles, and a lizard. A total of 431 *Blattella germanica *species were captured in 28 (9.96%) traps, and 84.2% of them were nymphs. One ship was highly infested. Cockroach infestation was negatively associated with ferries in which Hazard Analysis Critical Control Point system was applied to ensure food safety on board (Relative Risk, RR = 0.23, *p *= 0.03), and positively associated with ferries in which cockroaches were observed by crew (RR = 4.09, *p *= 0.007), no cockroach monitoring log was kept (RR = 5.00, *p *= 0.02), and pesticide sprays for domestic use were applied by crew (RR = 4.00, *p *= 0.05). Cockroach infested ships had higher age (*p *= 0.03). Neither rats nor mice were found on any ship, but three ferries had been infested with a rodent in the past.

**Conclusion:**

Integrated pest control programs should include continuing monitoring for a variety of pest species in different ship locations; pest control measures should be more persistent in older ships. HACCP system aids in the prevention of cockroach infestations on board.

## Background

Ships carry not only passengers and their luggage, vehicles and their loads, but may carry unwanted organisms such as flies, cockroaches, mosquitoes, fleas, and even other pests. These injurious organisms may enter a ship either directly from the ship's open spaces including ramps, portholes, or hawsers, they may be carried in food supplies, cargos, luggage, and vehicles, or may be carried on humans or animals as ectoparasites.

Vectors on board ships can cause harm in different ways. They may cause illness on board ships, and this may happen through the consumption of food containing human enteropathogens, mechanically transmitted by flies or cockroaches. Stored food products aboard ships may be damaged or contaminated by live or dead insects, faeces, odours, webbing or cast skins. Furthermore, vectors such as mosquitoes may be introduced, and established in areas in which they have not previously been found [1,2] and where vector borne diseases can spread. There are at least five reported cases of port malaria among people who had no recent travels or blood transfusions, but worked or lived close to harbours in Italy, France, Belgium and Israel [3-6]

Ferries serve as a means of transport on regular itineraries from one place to another and are becoming an increasingly popular form of travel in Greece. Among the EU ports, Piraeus registered an increase (+3.4%) of the number of passengers traveled in 2005 comparing with the previous year.{Amerini, 2007 312/id}. About 30 million passengers and 7 million vehicles are carried by ferries in Greece annually.

Ferries provide conditions suitable for the survival and growth of insect populations. Even though they are not the natural habitat conditions for many arthropod species, there are a variety of harborage areas and when sanitation measures are not adequately taken food sources are available for many synanthropic insects such as flies, cockroaches and ants. Inaccessible spaces including behind and below equipment, in voids and ducting, and even between bulkheads and deckheads, are difficult in treatment once infested. In addition, standing water at different areas of the ship open spaces like lifeboat covers, bilges, scuppers, awnings, gutters, and air treatment plants can hold insect larvae.

Closed living accommodation favour the spread of ectoparasites from one person to another. Historically, typhus fever was one of the diseases responsible for the high death rate among the crowded prison ships in the New York harbour during the American Revolution. Overcrowding, bad hygiene and lack of ventilation made a ship an ideal environment for typhus, which was passed on by lice [8]. In recent years, scabies outbreaks, a disease caused by mites, have been reported on board cruise ships (personal communication, EU SHIPSAN project Partnership, September 2007).

Various itineraries in ports of different countries, where no vector control measures are applied may increase the possibility of ship infestations. To our knowledge no studies exist on the examination of port itineraries in relation to ship infestation. Port areas are of high risk for infestations as, a large number of different types of ships arrive, load or unload cargoes originating from all over the world. In addition, great quantities of food are transferred and stored in storage areas of ports, while containers stacked in docks provide harbourage places for pests.

Although there are many published studies describing pest infestations of land-based establishments such as hospitals and restaurants [9-12], few data exist for the presence of arthropods and rodents on board passenger ships in resent years. The aim of this study was: a) to survey ferries for the presence of rodents and arthropods of public health importance, b) to identify ship locations which facilitate pest infestations, and c) to evaluate the implemented pest control programs.

## Methods

### Data collection

A detailed standardized questionnaire was developed to record information about ship characteristics and pest control practices, which were used to evaluate risk factors possibly associated with pest infestations. In particular, the questionnaire included information on: 1) ship characteristics: ship name, ship type, owner, itinerary, net tonnage, gross tonnage, ship age, number of beds in cabins, and passengers and crewmember capacity, 2) details of the certificates: date and results of the deratting/exemption deratting certificate (International Health Regulations, 1969), date of the last disinsection, method and pesticides used, recent environmental health inspection results in the inspection report, certificate for the application of HACCP, and 3) other information collected by the designated crew members such as: whether pests have been seen or captured on board in the past; pesticides applied by crew members, their frequency, method and formulation; and requests for insect repellents and after-bite insect treatment medicines by passengers.

A standardized form was used to record the number, the species and the life cycle stage (egg, nymph, adult) of pests captured in the traps which were placed on board, the ship location that each trap was placed, and the unique code of each trap.

In addition, the archives of the Piraeus Port Health Authority and the Department of Hygiene of the Ministry of Mercantile Marine were reviewed to identify complaints related to the presence of pests aboard ships from 1994 to 2004. Only complaints which were investigated and confirmed by the Public Health Officers were included in our study results.

### Sampling

The survey was conducted in 21 ferries. In order to survey ship locations for the presence of insects and rodents, two different types of traps were used: 1) the Lo-Line sticky glue trap (210 × 100 × 22 mm), which was used with 13 mm diameter tablets lures containing both food attractant and a yeast-based mimic of the aggregation pheromone used by cockroaches to enhance attraction, and 2) the TRAPPER 24/7 MULTI CATCH MOUSE TRAP, which was used together with a TRAPPER LTD glue board to monitor for insects and mice.

A standard number of traps ranging from 10 to 20 were placed in specific locations of each ship (galleys, bars, food storage rooms, passenger storage rooms, engine room, garage etc). The number of the traps placed in each ship was proportional to the size of the ship and the number of public health importance areas on board (e.g. number of galleys, food storage rooms etc.). Traps were coded and remained aboard ships for 7 to 10 nights. Traps with captured cockroaches were immediately transferred to the laboratory. Cockroaches were kept at refrigerator temperature.

The identification of the species was conducted in the Department of Parasitology, Entomology & Tropical Diseases of the National School of Public Health in Athens.

All ships continued to operate on their regular voyage schedules during the study period.

### Microbiological examination for *Salmonella *spp

Trapped cockroaches were investigated for *Salmonella *spp. They were killed by freezing at 0°C for 24 h. Cockroaches from each coded trap were placed in sterile tube with sterile normal saline and then thoroughly shaken for 2 min. A fixed volume of this washing was inoculated on Buffered Peptone Water (OXOID, Basingstoke, UK) and incubated at 37 ± 1°C for 18 h, and then subcultured in Rappaport-Vassiliadis (OXOID, Basingstoke, UK) (1:100) at 42°C for 24 h. Simultaneously, 10 μl of the solution were subcultured in Xylose lysine deoxycholate (XLD) agar and Brilliant Green agar at 37 ± 1°C for 18 h. Colonies were cultured on Kligler agar.

The microbiological examination was conducted in the Department of Microbiology of the National School of Public Health in Athens.

### Statistical analysis

Data were analyzed with Epi-Info 2000 (CDC, Atlanta, GA, USA) and SPSS for Windows Release 11.0.1 software (SPSS Inc, Chicago, IL, USA) by the Mann-Whitney test for quantitative data, and by chi square test or Fisher exact test for qualitative data. The median and the interquartile range (IQR) were used to delineate the dispersion of quantitative data. Relative risk (RR) and 95% confidence interval (CI) were calculated to assess categorical risk variables associated with insect infestations.

## Results

### Pest species

Species trapped on board during the survey, or had been found by crew members according to the data obtained through the interviews, or according to the passengers complaints recorded in the authorities' archives were: flies, cockroaches, ants, spiders, butterflies, beetles, bedbugs, fleas, a lizard, and rodents (Table 1). The most common pests sighted by the crew members on board were flies were. Out of the total 21 ships, 18 (85.7%) were infested by flies. In 11 ferries (52.3%) cockroaches were captured in traps which were placed on board. Complaints by passengers for the presence of bedbugs were reported on three ships (14.3%) and public health officers captured insects in the lounge of one ship. Complaints by passengers were reported for the presence of fleas for one ship (4.8%).

**Table 1:** Pest species trapped or had been found by crew members on board 21 ferries

Species	Number of individuals	Number of ships	Ship location	Source
*Blattella germanica*	431	11	Galleys, dinning rooms, bars	Trapped on board, complaints, crew interview
*Drosophila *spp.	15	3	Food storage rooms	Trapped on board
*Musca domestica*	Several	18	Galleys, dinning rooms, bars	Crew interview
Bedbugs^1^	Several	3	Lounge, cabin	Trapped on board^2^, complaints
Fleas^1^	Several	1	Cabin	Complaints
Ants^1,3^	17	2	Garage, food storage room	Trapped on board
Beetles (coleoptera)^3^	1	1	Garage	Trapped on board
Butterfly (lepidoptera)^3^	1	1	Garage	Trapped on board
Spiders^3^	2	2	Garage, luggage storage room	Trapped on board
Reptiles (*Chalcides ocellatus*)^1^	1	1	Cabin	Crew interview
Mice or rats ^1^	3	3	Galley,^4 ^garage, deck	Crew interview, complaints, other evidence^4^

^1^These species were not sent to the laboratory for identification.

^2^Trapped on board by Public health officers in 2003.

^3^Captured in rat-traps

^4^A rat trap was placed in the galley of a ferry that mainly transferred tracks and merchandise

### Cockroaches

About 52.3% (11/21) of the ferries had cockroach infestations. One ship was highly infested with 380 cockroaches; another 4 ships were infested with 6 to 13 cockroaches, while 6 ships had low infestation (less than 5 cockroaches).

A total of 431 cockroaches were collected from 11 ferries. A total of 298 traps were placed, and 17 traps were not found during the collection process. Cockroaches were captured in 28 (9.96%) traps out of the total 281 traps were collected. All cockroaches were *Blattella germanica *species, and 363 of the 431 (84.2%) were nymphs.

Infested locations included galleys, bars, pantries, food storage rooms, garbage, and dining rooms. The galleys were the most infested area of ships (in particular underneath refrigerators, Table 2).

**Table 2:** Ship locations with cockroach infestations

Ship location where traps were placed	Number of traps collected	Number of traps in which cockroaches were captured	Number of cockroaches trapped (% total)	Mean number of cockroaches
Galley – refrigerator	22	5	337 (78,19)	15,32
Galley – cooker	20	2	6 (1,39)	0,30
Galley – appliance	21	2	34 (7,89)	1,62
Galley – drawer	18	2	17 (3,94)	0,94
Bar – cash register	40	2	2 (0,46)	0.05
Bar – toaster	39	2	3 (0,70)	0,08
Pantry – refrigerator	7	1	9 (2,09)	1,29
Food storage room	42	7	7 (1,62)	0.16
Luggage storage room	13	-	-	-
Garbage	15	1	4 (0,93)	0,27
Dining room – refrigerator	37	4	12 (2,78)	0.31
Engine room	7	-	-	-
Garage	Not found	-	-	-
Deck (outdoors)	Not found	-	-	-
Location for pets (outdoors)	Not found	-	-	-

Total	281	28	431	-

*Salmonella *was not isolated from any cockroaches examined.

### Rats

A total of 205 rat traps were placed, but neither rats nor mice were found on any ship. According to the data collected through the crew member interviews, in one particular ship a rat was found in the garage, while for another ship complaints over the presence of a rat were reported to the Public Health Authority by passengers. During the survey, a rat trap with bait not set by us was found in the galley of an old ferry mainly used to transfer merchants and tracks, allowing us to believe that rodents must have been sited. Although crew members denied the presence of rodent, this ship was considered as infested (Table 1).

### Pest control methods

Two out of the 21 ships that participated in the survey were engaged on international voyages and proved to hold valid exemption deratting certificate as required under the International Health Regulations, 1969. Disinsection on all ships were conducted by qualified external companies on a regular basis.

Table 3 presents the results of the analysis conducted to identify association of the applied pest control practices on board with that of cockroach infestations. Using univariate analysis, cockroach infestations negatively associated with ferries that applied HACCP (*p *= 0.03), and positively with ships in which cockroaches were observed by the crew (*p *= 0.007), no cockroach monitoring log was kept (RR = 5.00, *p *= 0.02), and pesticide sprays for domestic use applied by crew (*p *= 0.05), (Table 3). Ship cockroach infestations were positively associated with the application of A and B pesticides of which the active ingredients were Cyfluthrin/spray (*p *= 0.01) and Fripronil/gel (*p *= 0.02) respectively.

**Table 3:** Association of applied pest control methods and practices, with cockroach infestations by univariate analysis

	**Ferries with cockroach infestations**		
	
**Factors**	**Factor Yes (%)**	**Factor No (%)**	**RR (95% CI)**
**Pest control methods and practices**			

Cockroaches observed by crew	9 (81.8)	2 (20.0)	4.09 (1.14–14.57)
Flies observed by crew	6 (60.0)	5 (45.5)	1.32 (0.58–3.00)
Regular visual inspections by crew	10 (58.8)	1 (25.0)	2.35 (0.41–13.45)
Use of spray pesticide by crew	10 (66.7)	1 (16.7)	4.00 (0.64–24.80)
Fumigation application	2 (40.0)	7 (58.3)	0.68 (0.21–2.22)
Absence of cockroach monitoring record log	10 (71.4)	1 (14.3)	5.00 (0.79–31.62)
Pesticide A: Cyfluthrin/spray	7 (87.5)	4 (30.8)	2.84 (1.20–6.96)
Pesticide B: Fripronil/gel	8 (80.0)	3 (27.3)	2.93 (1.06–8.08)
Pesticide C: Alphacypermethrin/spray	5 (83.3)	6 (40.0)	2.08 (1.01–4.26)
Pesticide D: Deltamethrin/spray	1 (50.0)	10 (52.6)	0.95 (0.22–4.05)
Pesticide E: Methylisobutylketone/spray	1 (50.0)	10 (52.6)	0.95 (0.22–4.05)
Pesticide F: Permethrin/smoke generator	2 (66.7)	9 (50.0)	1.33 (0.52–3.3)
**Other factors**			
Pet transportation on board	7 (53.8)	4 (50.0)	1.07 (0.45–2.53)
HACCP application	1 (16.7)	10 (71.4)	0.23 (0.03–1.43)
Itinerary: North-East Aegean	5 (83.3)	6 (40.0)	2.08 (1.01–4.26)
Itinerary: Cyclades	5 (55.6)	6 (50.0)	1.11 (0.49–2.50)

Table 4 presents the median and IQR of different ship characteristics in association with cockroach infested and non infested ships. Infested ships had a statistically significant higher age than those with no infestation (*p *= 0.03) (Table 4).

**Table 4:** Media and interquartile range (IQR) of ship characteristics in association with cockroach infestations

Characteristics	Ferries **with **cockroach infestations		Ferries **without **cockroach infestations		
	No of ferries	Median (IQR)	No of ferries	Median (IQR)	*p *value

Ship age	11	30 (25–31)	10	5 (5–5)	**0.003**
Days since last disinsection	11	40 (39–84)	9	63 (48–67)	0.2
Number of beds	11	308 (193–630)	10	362 (104–758)	0.7
Days since last Exception Deratting Certificate	7	138 (34–141)	8	79.5 (33–126)	0.9
Ship length (m)	11	137 (130–170)	10	157 (124–197)	0.3
Gross tonnage	9	9,124 (6,777–20,446)	9	7,895 (5,651–24,352)	0.6
Passenger capacity	11	1,500 (1,006–1,730)	10	1,645 (1,313–1,890)	0.3
Number of decks	10	7.5 (7–8)	9	8 (8–10)	0.1

## Discussion

Our study confirms the presence of different pests of public health importance on board ferries. The house fly was the most commonly found insect on board (85.7% of the ships), even though ferry galleys are mechanically ventilated and the open spaces where flies can enter a ship are limited. The second most frequently noted pest was the cockroach. Both synanthropic insects can significantly contribute to the spread of food-born protozoan diseases [13]. Cockroaches have been recognized as a major cause of asthma morbidity and several cockroach-produced allergens have been identified and characterized [14]. A study carried out in Hamburg, Germany, indicated that cockroach sensitized seamen often showed the symptoms of obstructive lung function impairment, which is evidence for an occupational respiratory risk due to the shipboard exposure to cockroaches {Oldenburg, 2006 616/id}. Inspection reports posted on the US Centers for Disease Control and Prevention, Vessel Sanitation Program website often include violations concerning the presence of cockroaches, drain flies, house flies and fruit flies aboard cruse ships [16].

Our study suggests that about 50% of the 21 ferries surveyed were infested with *Blattella germanica *and the majority of them had not great infestation. One ship was found to be highly infested; another 4 ships were moderately infested, while 6 ships had low infestation. The majority of the cockroaches collected were nymphs. This probably indicates that the adult population was eradicated through the application of pesticides, but nymphs were able to survive in their harbourage areas, where pesticides are difficult to apply.

The absence of *Salmonella *spp. from the cockroaches tested does not exclude the presence of other enteropathogens. Other studies carried out in different land based establishments have shown that various pathogenic organisms have been isolated from cockroaches' cuticle or gut [17,18].

In order to collect information on pest species which can be found on board ships generally, we used complaints data from the archives of Public Health Authorities. This was used to have an indication of the pest species and not to make any conclusions about the nuisance caused by them or the frequency of their presence and the size of their population on board. Very few people will complain about mosquitoes or other biting insects, but they may be on board and a public health threat to passengers and new areas.

No rodents were captured aboard the 21 ferries during the survey. It is possible that one old ferry was rodent-infested during the study, due to the presence of a trap which was placed in the galley by the crew members. According to the information collected through the interviews and the review of the Port Health Authorities archives, it seems that rodents can be found only occasionally on board, since over the past 10 years, only one complaint was reported by passengers and one sighting by a crew member.

There are few studies that report the extent of rodent infestations still occurring on ships. These studies that have been published, report upon merchant ships. Findings of surveys carried out by the Government of China Import and Export Inspection and Quarantine Department Bureau indicated that from 1990 to 1998, 24.7% of the 1093 incoming ships examined were infested with rodents [19]. Another study carried out in the Shimizu port, Japan reported that 47 rats captured aboard vessels. Genetic studies of the ship rats showed that they were different from the rat populations in the harbor [19].

Three ferries were infested with bedbugs. It is possible that bedbugs were transported in luggage or in bedding that were carried onto ships by passengers. The ferry environment provides areas suitable for bedbug harbourage: in cracks and crevices in furniture and walls, in upholstered furniture, and in mattress seams. The presence of bedbugs has increased dramatically in hotels and motels and on cruise ships, as well as in college dorms and nursing homes, over the last five to 10 years [20]. Furthermore, the incidence of skin disease secondary to infestation with the human bedbug, *Cimex lectularius*, has increased dramatically in the United States and in the United Kingdom [21]. Extermination of bedbugs using cleaning techniques and pesticides is necessary to prevent recurrence [22].

We report an isolated incidence of flea infestation in one ferry. Flea control is justified by the direct and indirect pathogenic roles of fleas (transmission of plague, tularaemia, myxomatosis, *Dipylidium caninum*) [23]. Since fleas are ectoparasites of dogs and cats, it is important to point out that those pets should be transported in specific ship locations especially designed for that purpose, while health certificates should be held.

Unfortunately, we were not able to study possible invasions of species found aboard ships to the destinations. This required knowing the species that are native or not in many different regions in Greece, but there are not enough data available for all these regions. It is possible that most of the species found such as German cockroaches, house flies, fleas and bedbugs are just being transported between regions where they are native already.

Ship itineraries did not show any association with ship infestations. This would be unlikely to happen because many ferries had the same destinations and similar itineraries, while the frequency of travel did not differ among them.

Our findings suggest that pest control programs should focus on different ship locations depending on the pest species. Cockroach and fly control measures should focus on galleys, especially refrigerators and other electrical appliances, and places where food is stored or handled including dining rooms, bars, pantries, garbage rooms and food storage rooms. Both cabins and lounges can be infested with bedbugs. The presence of other pests such as ants, beetles, butterflies, and spiders in the garage of the ferries indicates that different pests can have access to ships when in ports, through open ramps. Control measures should be taken on a regular basis, in order to prevent rats entering the ships. Examples of such measures include placement of rat guards in hawsers, closure of ramps when they are not in use, lighting of the ships' open spaces during the night, and installation of self-closing doors.

Our study indicates that older ships had increased cockroach infestations, and therefore pest control programs should be more persistent and should be applied on older ships more frequently. The application of an integrated pest control program is more effective when including record monitoring. Ferries implemented hazard analysis within their operations to ensure food safety presented less cockroach infestations. The legislation of HACCP and its application in ship galleys may contribute to the reduction of cockroach infestations. The occasional application of pesticide sprays for domestic use by crew members is not effective for cockroach control.

Even thought two pesticides were positively associated with cockroach infestations, we cannot draw any conclusions on their effectiveness. We were not able to evaluate the method of application of these pesticides (locations and frequency of application etc), or to examine other factors such as pesticide resistance.

The revised International Health Regulations (2005) is the global community's new legal framework against acute public health risks that can spread internationally. The importance of vector borne diseases transmission through ships on international voyages has been recognized under these Regulations, and provisions for vector borne diseases control have been included regarding competent port authorities, ship operators, and designated national authorities. Countries should establish programmes to control vectors at ports where ships on an international voyage arrive of depart [24]. The 2004 draft of the Guide to ship sanitation of the World Health Organization includes aspects regarding design, construction and operational control measures for insects and rodents, as well as rat proof construction. It is intended to be used as a base for the development of national approaches to controlling the hazards on ships, as well as providing a framework for policy making and local decision making. Adherence to the requirements of the revised International Health Regulations (2005) and to guidelines of the WHO Guide to Ship Sanitation is expected to contribute in eliminating the presence of vectors at ports and on board ships.

## Conclusion

Different pests of public health importance can be found on board ferries. Integrated pest control programs should involve control measures for a variety of pest species including flies, cockroaches, fleas, bedbugs, ants, and rodents. Early identification of their presence is important to avoid large infestations. Measures for cockroach control should be more persistent in older ships, while different ship locations should be monitored and treated depending on the pest species. HACCP system aids in the prevention of cockroach infestations on board.

## Competing interests

The author(s) declare that they have no competing interests.

## Authors' contributions

VM participated in the design of the study, carried out the data collection and sampling and drafted the manuscript. RA carried out the data collection and sampling and participated in the design of the study. KT and CH carried out the data collection and sampling. ASV carried out the identification of pest species and contributed to the laboratory examinations. Ch.Had. conceived of the study, and participated in its design and coordination and helped to draft the manuscript. JK participated in the design and coordination of the study. All authors read and approved the final manuscript.

## Pre-publication history

The pre-publication history for this paper can be accessed here:


